# Supplementary material for: Comprehensive Annotation and Functional Exploration of MicroRNAs in Lettuce
Source: Front Plant Sci. 2021 Dec 24;12:781836. doi: 10.3389/fpls.2021.781836 (PMC8739914; doi:10.3389/fpls.2021.781836)
Supplement: Supplementary file 3 [file Data_Sheet_9.ZIP › GSM2230752.plot/Lsa-miR397_Lsat_1_v5_gn_5_169160.1_829_TPlot.pdf]

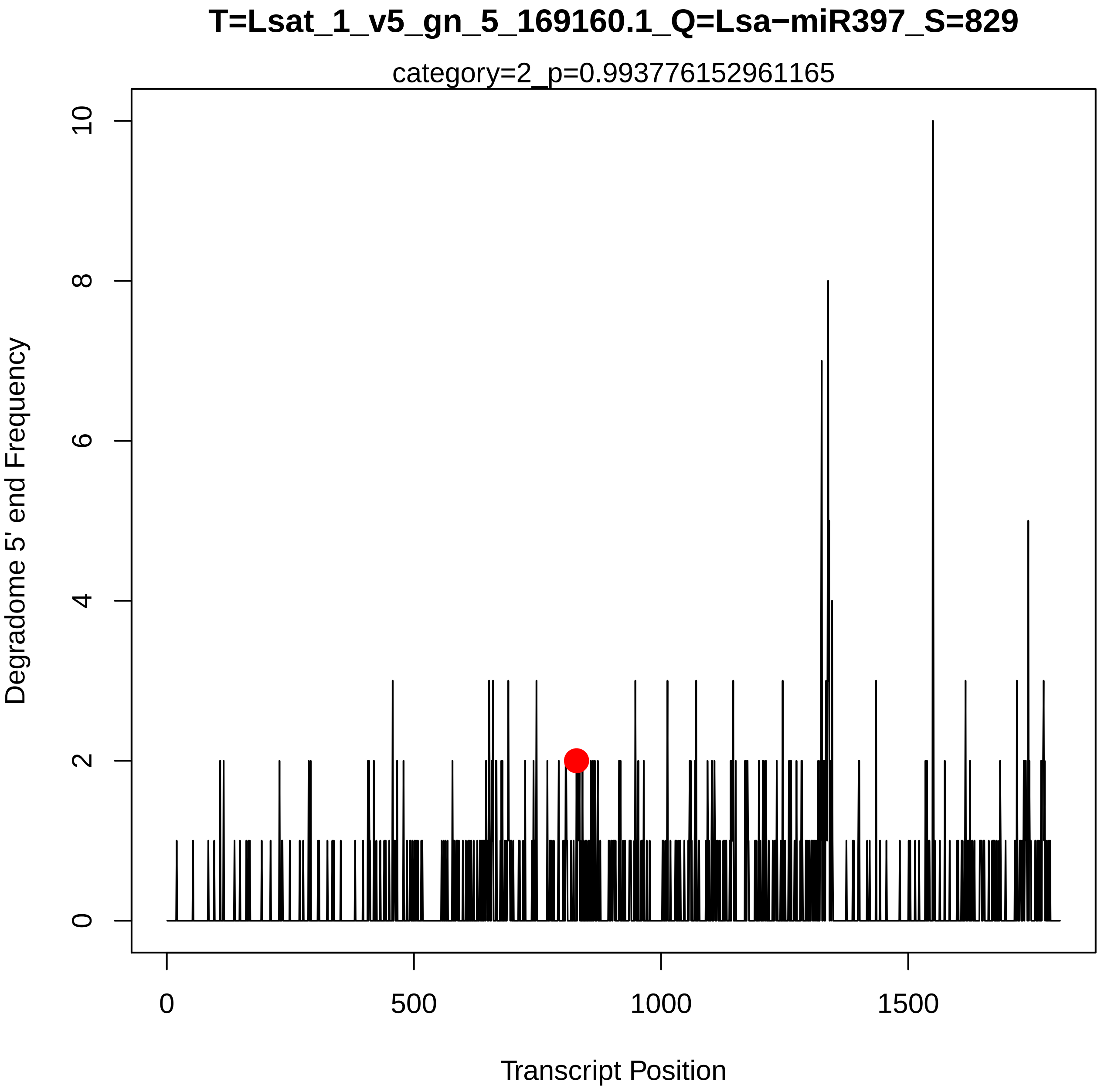

T=Lsat_1_v5_gn_5_169160.1_Q=Lsa−miR397_S=829
category=2_p=0.993776152961165
Degradome 5' end Frequency
0
2
4
6
8
10
0
500
1000
1500
Transcript Position
